# Supplementary figures and images for: Persistently high TB prevalence in Nairobi County neighbourhoods, 2015–2022
Source: PLOS Glob Public Health. 2025 Feb 18;5(2):e0003849. doi: 10.1371/journal.pgph.0003849 (PMC11835327; doi:10.1371/journal.pgph.0003849)

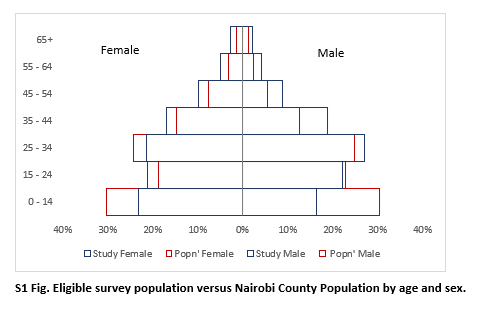

Supplement: S1 Fig — (TIF) [file pgph.0003849.s001.tif]
