# Supplementary material for: Persistently high TB prevalence in Nairobi County neighbourhoods, 2015–2022
Source: PLOS Glob Public Health. 2025 Feb 18;5(2):e0003849. doi: 10.1371/journal.pgph.0003849 (PMC11835327; doi:10.1371/journal.pgph.0003849)
Supplement: Attachment — Submitted filename: Response to Reviewers.pdf [file pgph.0003849.s010.pdf]

N. Sarita Shah, MD, MPH
Academic Editor
PLOS Global Public Health

November 26, 2024

Dear Dr. Shah,

We thank you and the reviewers for your careful attention to our manuscript, "Persistently high TB prevalence in Nairobi County neighbourhoods, 2015-2022." We are pleased to submit a revised version of the manuscript, which includes a point-by-point response to the reviewers' comments. Our responses are in red. We show changes to the manuscript text in **bold**.

Kind regards on behalf of all study authors,
Jane Rahedi Ong'ang'o and David Horne

Reviewer 1

1. Contributing Factors: The article would benefit from a more in-depth analysis of the underlying causes behind the persistently high TB prevalence in the nine clusters studied. It would be valuable to explore factors such as overcrowding, poor ventilation, poverty, and limited access to healthcare in these neighborhoods. This deeper understanding could help identify more targeted interventions and address the root causes driving TB transmission in these high-risk areas.

We appreciate these suggestions from the reviewer. We agree these are important risk factors for TB. While we noted the increase in unemployment as a potential factor, our study did not collect data on ventilation and access to healthcare. We have described the need for this future work in lines 427 -429.

Future work should explore factors such as overcrowding, poor ventilation, poverty, and limited access to healthcare in these neighborhoods to help identify more targeted interventions to address the root causes driving TB transmission in these high-risk areas.

2. Comparative Analysis with Other Regions: Including a comparative analysis between Nairobi and other urban centers in Kenya could provide broader context. Is TB prevalence also high in other cities, or is Nairobi an outlier? This comparison could help identify whether the issue is unique to Nairobi's urban dynamics or part of a larger regional trend. Lessons from cities with lower TB rates could offer strategies for mitigating the prevalence in Nairobi's clusters.

Thank you to the reviewer for this comment and we agree that broader context on Nairobi's estimated prevalence and insights into drivers of differences in burden could be gleaned from this . Unfortunately, the 2015 survey was not focused on subnational estimates and the 2022 survey was limited to nine Nairobi county geographic clusters. This may present opportunities to re-examine the 2015 data using novel methods for sub-

national estimates but this would be beyond the scope of the current study. We focused on Nairobi county because it reported the highest numbers of TB notification in the country.

We have added the following to the Discussion (limitations):

The study focus on Nairobi County does not allow for comparisons with other regions of the country. However, in addition to the findings from the prevalence surveys, there is indirect evidence of regional differences in Kenya. The annual risk of TB infection (ARTI) in Kenya was last described in 2004, at which time Nairobi District had the highest point estimate suggesting lesser impacts of TB control efforts on transmission compared to other districts at that time.[34] (lines 420-424)

3. Impact of COVID-19 and the Kenyan Health Worker Strike: The article briefly mentions the effects of COVID-19 and the health worker strike on TB detection and treatment. A more detailed exploration of how these events impacted TB case detection, treatment adherence, and overall healthcare access would be beneficial. Such analysis could provide insights into how health system disruptions contribute to TB burden, particularly in urban settings like Nairobi.
Thank you for this suggestion. Our study design was cross-sectional and we were not, unfortunately, able to evaluate the impact of these temporal trends on our estimate. However, we do site a time series from Kenya that evaluated the pre-pandemic, early pandemic, later pandemic and health care worker strike time periods (citation 21: Kiarie, The COVID-19 pandemic and disruptions to essential health services in Kenya: a retrospective time-series analysis. Lancet GH 2022).

We added the following to the Discussion describing the impact of the pandemic on TB notifications (lines 353-356).

The number of notified tuberculosis cases was one of many Kenyan key health indicators that worsened with the onset of the pandemic, decreasing by 26·6% (14·7–45·1%) from March to April 2020.(lines 354 – 356)

4. Data Visualization: To enhance the clarity and impact of the findings, the inclusion of data visualizations, such as heat maps showing TB prevalence across the nine clusters, would be highly useful. These visual tools would allow readers to quickly grasp spatial and temporal trends in TB prevalence and would be particularly impactful when presenting the data at conferences or public health forums.

We appreciate this suggestion. Supplementary Table 4 provides the point estimate of each cluster crude prevalence.
(**S4 Table. Participants diagnosed with TB in 2022 and 2015 by geographic cluster.)**
The cluster neighborhood boundaries are not contiguous, and so it is unclear how to include unsampled areas in a heat map. We are exploring geospatial modelling approaches to estimate TB prevalence within Nairobi using these data.

Reviewer 2

- Overall, the paper is well-written. The author clearly outlines the rationale and objectives of the paper, which is to evaluate the prevalence of TB in Nairobi County through the serial surveillance of TB using a population-based survey. The discussion section can be strengthened by stating the local drivers of consistently high TB prevalence in Nairobi, as indicated by the survey. Also, the author may provide examples of evidence-based interventions that have proven effective to reduce the prevalence of TB in similar settings. This is helpful information to help the reader gain a better understanding of the public health implications of the paper's findings.

Thank you for your kind comments and helpful suggestions. Unfortunately, we were not able to have an in-depth analysis of the social and environmental risk factors between the 2 studies because this information was not equally available to support comparisons. We have added the following to the Discussion (lines 427-429):

Introduction:

1. The author's hypothesis statement, lines 68-69, is not clearly written. Please revise, as it seems that part of the sentence was cut out.

Thank you for requesting this clarification. We have re-phrased out hypothesis with the following (

We hypothesized that TB prevalence would decrease between 2015 and 2022 in the setting of accurate TB estimates from the 2015 survey, increased case notifications since the prior survey, and greater availability of GeneXpert tests for rapid TB diagnosis.(lines 68-70)

Methods:

1. In line 97 where it reads 'The household-level survey procedures were similar for both surveys', please cite the protocol for the 2015-2016 TB prevalence survey or another publication where the survey procedures methods have been summarized. When referring to methods explained elsewhere, please include a citation so that readers may refer to that work.

Thank you. We have clarified this by adding a citation [3] for reference, line 99

2. Can you explain why information on diabetes was not collected in the 2015-2016 survey?

Unfortunately, the 2015 survey data collection tool did not include information on diabetes and we are unable to identify people who might have insight as to why questions on diabetes were not included in that survey.

Discussion:

1. In line 330, it is mentioned that 'local drivers of the TB epidemic are stable and may be used to develop targeted TB control interventions.' Did the survey highlight what these drivers may be? It is helpful to clearly state what the results show to be the local drivers

of TB and provide examples of interventions, supported by the literature, that may successfully reduce TB prevalence in this context.

Thank you for these comments. In our discussions we have explained high-risk sub-populations (e.g. men, ages 45-54 years) (lines 329 – 331)and possible local scenarios that may have contributed to the persistently high TB prevalence such as the COVID _19 pandemic and the health care worker's strikes which disrupted TB services (lines 351 - 354).

We compared sociodemographic variables between the 2 surveys ,we found there was increased unemployment in 2022 that was statistically significant this may be a contributing drive to TB disease, we have explained this in lines 360-361. Unfortunately our comparison analysis was limited to information that was in both the 2015 and 2022 surveys.

Regarding interventions, the evidence is mixed in regards to strategies to decrease community burden of TB and we hope that our study contributes to the call for funding of these types of studies. We added the following to the discussion (lines 383-389):

However, it is important to note that we likely missed participants with TB who were without cough or abnormal chest x-ray as we would not have collected sputum from these participants. There is growing recognition of the importance of sub-clinical or asymptomatic TB as a large proportion of prevalent TB and as a likely driver of ongoing TB transmission.[29, 30] Achieving early diagnosis and treatment in persons with TB without symptoms (with or without radiographic abnormalities) will require novel methods to address diagnostic gaps including point-of-care testing, high population diagnostic yield, and relevance to active case-finding.[2] (lines 381-387)

Ethical considerations

This has been expanded to describe the informed consent process and participant confidentiality (lines 189 -199)

The sub-scetion `Inclusivity in Global Research' was added in lines 200 -202 as recommemded.

References

The following new references were added;

29. Emery JC, Dodd PJ, Banu S, Frascella B, Garden FL, Horton KC, et al. Estimating the contribution of subclinical tuberculosis disease to transmission: An individual patient data analysis from prevalence surveys. Elife. 2023;12.

30. Horton KC, McCaffrey T, Richards AS, Schwalb A, Houben R. Estimating the impact of tuberculosis pathways on transmission - what is the gap left by passive case-finding? J Infect Dis. 2024.

These 2 references describe the growing recognition of the importance of sub-clinical or asymptomatic TB as a large proportion of prevalent TB and as a likely driver of ongoing TB transmission.

Another added reference was;

34. Kwamanga D, Chakaya J, Sitienei J, Kalisvaart N, L'Herminez R, van der Werf MJ. Tuberculosis transmission in Kenya: results of the third National Tuberculin Survey. Int J Tuberc Lung Dis. 2010;14(6):695-700.

This reference informs on the 2004 annual risk of TB infection (ARTI) in Nairobi being the highest in the country
